# Detection of delirium by nurses among long-term care residents with dementia

**DOI:** 10.1186/1472-6955-7-4

**Published:** 2008-02-26

**Authors:** Philippe Voyer, Sylvie Richard, Lise Doucet, Christine Danjou, Pierre-Hugues Carmichael

**Affiliations:** 1 Faculty of Nursing, Laval University, Quebec City, Canada; 2 Laval University Geriatric Research Unit, St-Sacrement Hospital Centre, Quebec City, Canada; 3 Long-term care department, Centre de santé et de services sociaux de la Vieille-Capitale, Quebec City, Canada; 4 Faculty of Nursing, Laval University, Quebec City, Canada; 5 Center for Excellence in Aging, St-Sacrement Hospital Centre, Quebec City, Canada

## Abstract

**Background:**

Delirium is a prevalent problem in long-term care (LTC) facilities where advanced age and cognitive impairment represent two important risk factors for this condition. Delirium is associated with numerous negative outcomes including increased morbidity and mortality. Despite its clinical importance, delirium often goes unrecognized by nurses. Although rates of nurse-detected delirium have been studied among hospitalized older patients, this issue has been largely neglected among demented older residents in LTC settings. The goals of this study were to determine detection rates of delirium and delirium symptoms by nurses among elderly residents with dementia and to identify factors associated with undetected cases of delirium.

**Methods:**

In this prospective study (N = 156), nurse ratings of delirium were compared to researcher ratings of delirium. This procedure was repeated for 6 delirium symptoms. Sensitivity, specificity, positive and negative predictive values were computed. Logistic regressions were conducted to identify factors associated with delirium that is undetected by nurses.

**Results:**

Despite a high prevalence of delirium in this cohort (71.5%), nurses were able to detect the delirium in only a minority of cases (13%). Of the 134 residents not identified by nurses as having delirium, only 29.9% of them were correctly classified. Detection rates for the 6 delirium symptoms varied between 39.1% and 58.1%, indicating an overall under-recognition of symptoms of delirium. Only the age of the residents (≥ 85 yrs) was associated with undetected delirium (OR: 4.1; 90% CI: [1.5–11.0]).

**Conclusion:**

Detection of delirium is a major issue for nurses that clearly needs to be addressed. Strategies to improve recognition of delirium could result in a reduction of adverse outcomes for this very vulnerable population.

## Background

Delirium is defined as a mental disorder of acute onset with a fluctuating course, characterized by disturbances in consciousness, attention, orientation, memory, thought, perception and behaviour [1]. It is a common problem among elderly patients admitted to acute and long-term care (LTC) facilities, with its prevalence ranging from 9.6% to 89% [2-4]. The wide variation in these estimates is likely related to methodological issues such as differences in the population under study, the type of clinical settings in question and the criteria used for diagnosis. For example, higher prevalence was found in cohorts composed of hospitalized elderly patients with pre-existing cognitive impairment [5,6].

It is now recognised that delirium in older patients is associated with numerous negative outcomes. Consequences for elderly patients who develop delirium include decline in their functional and cognitive status as well as increased morbidity and mortality rates [7-10]. Furthermore, older patients with delirium have been found to be at greater risk for pressure ulcers, falls and pneumonia [11]. Deleterious effects of delirium are not limited to patient outcomes. It also represents a great source of distress for family members, an increased nursing time per patient and higher costs for hospitals [12]. Moreover, since delirium can be an early indication of an underlying medical condition such as sepsis or a myocardial infarction, not recognising it may prove detrimental for the patient who may then become critically ill [13].

Early recognition of delirium allows not only prompt management of the underlying medical cause but also permits the rapid implementation of targeted interventions against predisposing and precipitating individual risk factors, thereby resulting in a reduction of delirium severity, duration and consequences [3,14-18].

Given its high prevalence rates, associated complications and potential underlying medical emergency, delirium and its detection should be of major concern for clinicians and especially for nurses, who spend more time at the patient's bedside.

Despite its clinical importance, delirium among older hospitalized patients often goes undetected. Several studies have studied the delirium detection rate of nursing staff in different clinical settings and recognition rates as low as 31% have been reported [3,19,20].

Some studies have identified factors associated with delirium undetected by nurses in hospital settings and although their results cannot be generalized to older residents in LTC settings, a review of these is worthwhile. These factors can be divided into three groups: factors related to the characteristics of: 1) the delirium itself, 2) the elderly, and 3) the bedside nurse.

Two delirium-related factors have been linked to delirium that is undetected by nurses among elderly hospitalized patients: its forms and its fluctuating nature. Delirium can be classified into four different subtypes depending on its clinical manifestation: the hypoactive form (characterized by lethargy, slow motor reaction, reduced interaction with surroundings), the hyperactive form (characterized by restlessness, hyper-vigilance and aggressiveness), a combination of both (mixed) or the absence of both (normal motor pattern). Because patients with the hypoactive form of delirium are less likely to demonstrate behavioural disturbances interfering with nursing care than are patients with the hyperactive form, the hypoactive form often goes unrecognised by nurses [19,20]. Since the hypoactive form is more prevalent among older patients [1,11], such non-recognition constitutes an important issue. In addition to this, the fluctuation of symptoms during the course of the day that is characteristic of episodes of delirium, makes its detection even more challenging [1]. In this sense, delirium may go undetected if the evaluation is not based on observations gathered over a sufficient period of time.

With regard to patient characteristics, three factors have been associated with the delirium undetected by nurses. Inouye et al. [20] have shown that having impaired vision, being over 80 years of age and pre-existing cognitive impairment are all factors linked to increased risk of under-recognition. The importance of prior cognitive impairment for under-recognition of delirium was further confirmed in a study by Fick and Foreman [5]. Although dementia and delirium are two distinct entities, the overlap of some of the symptoms may make delirium detection more difficult, especially if the individual assessing it lacks proper training.

Undetected delirium has also been found to be linked to nurse-related factors such as a lack of knowledge about delirium and how to detect it [3,21]. For example, nurses were found to frequently use the term "confused" and "acute confusion" inappropriately [22]. Furthermore, the lack of awareness among the nursing profession of the clinical importance of delirium was also found to be involved in this situation [14].

In addition to the aforementioned factors, some authors have argued that under-recognition of delirium likely reflects a poor conceptualization of delirium and consequently scientists in general should also take some ownership of the problem [23].

As already mentioned, most studies on the detection of delirium have been carried out in hospital settings. Expanding our knowledge about delirium and the way nurses go about detecting it in elderly residents in LTC facilities, is essential to being able to offer quality health care to this population. Moreover, given the high prevalence of dementia in LTCS, efforts should be made to target this very frail and unique population. To the best of the authors' knowledge, there has been no study to date examining the detection rate of delirium and its associated factors among seniors with pre-existing dementia residing in LTC facilities. As the general population ages, the prevalence of delirium among this specific population is likely to increase [14], as is the risk of under-recognition of delirium and this underscores the need to fill this gap in our knowledge. The objectives of the present study are:

1) To determine the rates for nurse-detection of delirium among seniors with pre-existing dementia in LTC facilities.

2) To identify those delirium symptoms that are most challenging for nurses to detect.

3) To identify those factors associated with delirium that goes undetected by nurses among this specific population.

## Methods

### Study design

This is a prospective clinical study with 2 repeated measurements at a time interval of 7 days (T1 and T2). The rationale for the study design was to look at temporal variability in order to study predisposing and predicating risk factors for delirium, something which is the objective of a future article.

### Study settings and selection of participants

Subjects were recruited in three LTC facilities and one LTC unit of a large regional hospital, all located in the Quebec City area, Canada. A convenience sampling procedure was used. Residents with the diagnosis of dementia, aged 65 years and more, and with no history of psychiatric illness, were eligible for this study. Following all appropriate institutional ethics committee clearances, a research assistant met with the head nurse from each participating facility so as to identify eligible residents. Specifically, the unit head nurse was asked to use resident chart information to identify residents with a diagnosis of dementia.

Following identification of eligible residents, there was an informative letter about the study sent to the families of said residents, inviting them to contact the research assistant to obtain further information, if so desired. Once their questions had been satisfactorily answered, those who had contacted the research assistant were then invited to sign proxy consent forms. As well, the research assistant obtained assent for participation from those residents whose cognitive impairment was judged to be mild or moderate.

In total, there were 293 families of demented residents solicited. Of these, 122 did not respond (41.6%), 11 refused to take part (3.8%) and 160 agreed to give their consent (54.6%). Following enrolment, three residents died before the first evaluation and one was transferred, leaving a total of 156 participants at T1. One resident was hospitalized at T2 and was therefore excluded from the analyses.

The nurses of the residents were also invited to take part in the study and their consent also obtained. A total of 40 nurses participated in the study.

### Data collection

The research assistants were nurses who had completed 15 hours of instruction on delirium and dementia, given by a member of the research team. Instructions on the research procedures as well as direct supervision in the data collection for 15 participants were also provided. All observational measures were completed based on resident monitoring over a seven-hour period at T1 and T2. To avoid potential bias, there were two research assistants involved in this study. The first (RA-1) focused on completing the measures of delirium (forms and severity) and dementia (type, stage and severity). The second (RA-2), was blinded to each resident's delirium status, and conducted a standardized interview with the bedside nurses in order to gather the following information: the bedside nurse's rating of delirium (overall and symptoms-specific) and some baseline characteristics of the nurse (age, sex, level of education, years of experience as a nurse and years of experience in geriatrics). RA-2 also collected basic demographic data (age, sex, marital status, level of education, ethnicity) and health information on the resident (number of days since admission to LTC setting, behaviour problems, functional autonomy, comorbidity, pain, depression, sleep problems, visual and hearing impairment, dehydration, weight loss, brachial perimeter, geriatric fever, oxygen saturation, number of prescribed medications and the presence of physical restraints during the day). Some information was extracted from the medical chart and other information was observed or measured using specific instruments. It should be noted that the bedside nurses in our study received no specific instruction on dementia or delirium from the researchers.

### Procedure

At T1, RA-1 assessed the presence of delirium (using the *Confusion Assessment Method *[CAM]) and delirium symptoms (using 6 items of the *Minimum Data Set-2 *[MDS-2]. These measures were considered as the reference standard in analyses on sensitivity and specificity. During the seven-hour observation period, RA-1 had to perform at least three formal assessments of symptoms of delirium (e.g. attention, level of consciousness) by interviewing the residents; this before going on to complete the CAM at the end of observation period. At the end of the day, RA-2 then interviewed the bedside nurse and asked this latter to rate evidence of: 1) delirium (using one question) and 2) delirium symptoms (using the same 6 *MDS-2 *items), based on clinical judgment and observation made throughout the day. This same procedure was repeated at T2.

### Outcome variables

For the first two objectives of this study (detection rates of delirium and delirium symptoms by bedside nurses), sensitivities, specificities, positive and negative predictive values were computed. For the third objective, (factors associated with undetected delirium), delirium that was undetected by nurses at T1 was used as the outcome.

### Definition of study variables

#### Delirium and delirium symptoms

The *Confusion Assessment Method (CAM) *is an established and widely used test to assist with detection of delirium [24]. This instrument has been shown to be sensitive (94% to 100%) and specific (90% to 95%) compared to the diagnosis of a psychiatrist [24]. The tool assesses the 9 criteria for delirium specified in the revised third edition of the Diagnostic and Statistical Manual of Mental Disorders (DSM III-R), i.e. (1) acute onset and fluctuation of symptoms over the course of the day; (2) inattention (3) disorganized thinking, (4) altered level of consciousness; (5) disorientation; (6) memory impairment; (7) perceptual disturbances; (8) psychomotor agitation or retardation; and (9) altered sleep-wake cycle. The presence of criteria 1 and 2 plus the presence of either criterion 3 or 4 is indicative of a definite delirium.

For a diagnosis of probable delirium [3], the first criterion changes to *"acute onset ****or ****fluctuation of symptoms over the course of the day" *and the rest of the algorithm remains the same. In the present study, subjects who met the criteria for definite or probable delirium were defined as having delirium. This decision was based on the fact that a valid evaluation of acuteness of onset would have required an investigation in greater depth by the RA-1, which was not feasible in the context of the present study. In fact, at time 1, RA-1 had no knowledge of the prior status of the residents. This strategy has been used by other researchers of the field [3,25]. Inter-rater agreements for definite and probable delirium, using data from 27 participants (17% of the sample), were satisfactory, respectively, kappa of 0.60; 95% CI: 0.24–0.97 and Delta of 0.64; 95% CI: 0.47–0.81.

One question was used to measure detection of delirium by the bedside nurses: " *Based on your clinical judgment and observations, how do you rate the resident overall mental status?: 1) as usual; 2) more alert then usual; 3) more confused than usual; and 4) in delirium*". Scores of 2, 3 and 4 were considered indicative of detection of delirium. A previous study on detection [2] was the source of inspiration for this procedure.

*Delirium symptoms *were assessed using 6 items from the *Minimum Data Set-2 *(MDS-2) [26]. This instrument was initially designed to be used in all nursing home facilities in the United States of America to collect comprehensive and standardized data in order to improve health care for this specific population. The 6 items selected assessed the following delirium symptoms: easily distracted, periods of altered perception, disorganized speech, periods of restlessness, periods of lethargy and mental function that varies over the course of the day. Each symptom is scored as 0 = not present, 1 = present but not of recent onset, or 2 = present and appears different from usual level of functioning. In this study, subjects were defined as having a delirium symptom if they had a score of 1 or 2 for that specific item. A score of 1 was considered positive in addition to 2 for the reason previously mentioned: the difficulty posed for RA-1 in obtaining a valid measure of "acuteness of onset"[25].

#### Factors potentially associated with delirium undetected by nurses

In addition to baseline characteristics of both the residents (age, sex, marital status, level of education, days since admission to LTC setting) and bedside nurses (age, sex, level of education, years of experience as a nurse and years of experience in geriatrics), the following variables were also considered in analyses focusing on factors potentially associated with undetected delirium. They were all measured the same day.

*Delirium Severity *was assessed using a slightly modified version of *Delirium Index *(DI) [27]. The original version of the *DI *assesses the severity of 7 symptoms of delirium (inattention, disorganized thinking, altered level of consciousness, disorientation, memory impairment, perceptual disturbances and psychomotor agitation or retardation). The modified version comprised eight instead of seven items; the item "psychomotor activity" being divided into two items ("hypoactivity" and "hyperactivity"). This decision was based on the fact that psychomotor activity can fluctuate over time and the *DI *was completed based on observations over a seven-hour period. The research assistant rated the severity of each symptom on a 4-point scale: 0 = absent, 1 = mild, 2 = moderate and 3 = severe. The *DI *has been shown to have adequate inter-rater reliability (concordance coefficient: 0.77–0.93) and criterion validity (Spearman's correlation coefficient : r of 0.84) [27]. This variable was classed into 3 different groups according to levels of severity: an overall score of 7 and less was considered as mild; 8 to 14 as moderate and of 15 and higher severe.

*Forms of delirium*: subjects were classed into 4 different subtypes according to Meagher's criteria [28]: 1) Hypoactive (presence of 4 or more of the following: unawareness, decreased alertness, sparse or slow speech, lethargy, decreased motor activity, staring and apathy), 2) Hyperactive (presence of 3 or more of the following: hyper vigilance, restlessness, fast or loud speech, anger or irritability, combativeness, impatience, uncooperativeness, swearing, singing, laughing, euphoria, wandering, easy startling, distractibility, nightmares, persistent thoughts), 3) Mixed: must have satisfied both hypoactive and hyperactive conditions and 4) Without a motoric component: subjects exhibiting no motoric behaviours, as defined above.

*Dementia status *was ascertained by the presence of a medical diagnosis of dementia in the medical chart. *Stage of dementia *was determined by observation using the 7 stage of the Functional Assessment Staging; a valid and reliable instrument [29]. *Dementia severity *was appraised using the *Hierarchic Dementia Scale (HDS) *[30], consisting of 20 subscales assessing a broad spectrum of cognitive abilities. The subscales in turn, comprise either 5 or 10 items in decreasing order of difficulty. The maximum score for the entire scale is 200 points. Older adults who are cognitively intact generally achieve the maximum or close to the maximum number of points [31]. On the other hand, the scale displays almost no floor effect. Even those patients with severe dementia are often able to answer or react correctly to the easiest items on the lower end of a subtest [32]. The validity and reliability of the scale are well established [33]. This variable was treated in categories (mild, moderate, and severe) using tertiles as the cut-off point.

*Pain *was assessed using the DOLOPLUS-II [34]. Each of the ten DOLOPLUS-II items (somatic complaints, protective body postures adapted at rest, self-protection of sore areas, expression, sleep pattern, washing and/or dressing, mobility, communication, social life, behavioural problems) is rated on a 0–3 scale with higher scores indicative of increased difficulties due to pain. The validity and reliability of this measure has been supported in the literature [35,36]. A score of at least 5 out of 30 (the maximum pain score) is regarded as indicative of pain.

*Depression *was assessed using the *Cornell Depression scale *[37] which was designed to assess symptoms of depression among older adults suffering from dementia. It consists of 19 items corresponding to five dimensions (mood-related signs, behavioural disturbance, physical signs, cyclic functions, ideational disturbance). In this study, the assessor, RA-2, indicates if the symptom is absent (0), mild to intermittent (1) or severe (2). The scale has good test-retest reliability (*r *= .75) and excellent internal consistency (α = .83) [38]. Inter-rater reliability and concurrent validity are also well established [37,38]. A value of 6 and higher is indicative of depression [39].

*Comorbidity *was assessed by chart review using the validated and reliable *Charlson Comorbidity Index *(CCI) [40]. The overall score ranges from 0 to 37 with higher scores indicating greater comorbidity. A value of 8 and higher was the cut-off point.

*Behaviour problems *were assessed using the *Nursing Home Behaviour Problem Scale *(NHBPS) [41]. The NHBPS is a 29-item questionnaire measuring serious behavioural problems in nursing home residents. This scale has shown good inter-rater correlation and a high level of convergent validity [41]. The assessor (RA-2) is asked to report the frequency of each behaviour using a 5-point frequency of occurrence scale (0 = never, 1 = sometimes, 2 = often, 3 = usually, 4 = always). In the present study, residents were considered as having a behavioural problem if they scored at least 3 on the frequency occurrence scale for at least one item.

*Functional autonomy *of the resident was measured according to the *Functional Autonomy Measurement System *(SMAF) [42]. This 29-item scale measures functional ability in five areas: activities of daily living (ADL: 7 items), mobility (6 items), communication (3 items), mental functions (5 items) and instrumental activities of daily living (IADL: 8 items). For institutionalized subjects, a modified 20-item version (excluding the 8 IADL items as well as one item related to exterior mobility) has been developed. The disability for each item is scored on a 5-point scale: 0 = independent, -0.5 = with difficulty, -1 = with supervision, -2 = with help and -3 = dependent. This version of the scale has shown good test-retest and inter-rater reliability (ICC of 0.95 and 0.96 respectively)[43]. Higher score is indicative of severe autonomy impairment.

*Sleep problems *were measured using the *Insomnia Severity Index *(ISI) [44]. Each item of this 7-item instrument is rated on a 0–4 scale and the total score ranges from 0 to 28, with higher score indicative of severe insomnia. The internal consistency and concurrent validity of this scale have been demonstrated [44]. In this study, sleep problems was defined as having an overall score of 8 or more.

Presence of *physical restraint *was defined as the use of at least one physical restraint during the course of the day. Physical restraints include ties, straps or belts (which can be tied to the legs, ankles, arms, or waist), jackets, gloves, geriatric chairs equipped with security tables, or other devices designed to limit the mobility of the older person and over which the resident has no control. Half-bedrails, half doors, and unit locked doors forming a barrier or obstacle to keep the older person in a given area were not considered physical restraints.

*Visual and hearing impairment *were measured with 2 items from the SMAF [42]. Subjects scoring -0.5 (or less) for each item were considered as being impaired with regard to the particular sense. *Oxygen saturation *was measured with a pulse oximeter. An oxygen saturation of < 95% was considered to be abnormal [45,46]. Subjects were considered as having *geriatric fever *if they had an oral temperature of ≥ 99°F (37.2°C) or a rectal temperature of ≥ 99.5°F (37.5°C) [47]. *Hydration *was measured by evaluating the amount of liquid absorbed by the resident during the course of the day (7 hours observation). An amount of 500 ml or less was chosen as the cut-off point as an indicator of dehydration. Weight loss of ≥ 3 kg over the previous 6 months, as well as a brachial perimeter of ≤ 21 cm, were used as indicators of *malnutrition *[48]. From the medical chart, RA-2 extracted the *number of medications taken *by the resident.

### Statistical analyses

Our study was not of simple random design. In fact, every resident of each LTC facility who was eligible for the study was included in our sample. Nurses were not randomized on the residents either; they were the regular bedside nurses for those residents. In such a situation, it was important to verify that our design did not unduly inflate variance estimates in the planned analyses. Therefore the design effect was computed and was found to be very close to 1 (0.90–0.99), which means that the design of our study performs as if the resident-nurse pairs had been selected randomly.

First, simple descriptive analyses were used to describe the study population (residents and bedside nurses). Then, bedside nurse ratings of delirium (one question) were compared with RA-1 ratings of delirium, the reference standard. Sensitivity, specificity, positive predictive value (PPV), and negative predictive value (NPV) with their 95% confidence intervals were computed using standard formulas for each time measurement (T1 and T2). This procedure was repeated to compare bedside nurse ratings of individual delirium symptoms (6 MDS items) with RA-1 ratings of the same delirium symptoms (6 MDS items). The decision to collect information on bedside nurse ratings of individual delirium symptoms using the MDS-2 was made after the beginning of the study. This meant that 31 residents were excluded from these analyses because of missing values.

To evaluate whether or not certain factors were associated with delirium that was undetected by bedside nurses, logistic regression analyses were performed. The outcome of these analyses was undetected delirium by bedside nurses at T1. Bivariate analyses were conducted to assess the crude association between each of the independent variables considered and undetected delirium. A significance level of 0.1 was used to select variables for subsequent multivariate analysis [49]. All analyses were carried out using SAS for Windows, version 9.1.

It is worth noting that for two variables, forms of delirium and the age of the nurses, quasi-complete separation of the data was observed. This situation arises when, for example, nurses from one age group never detected delirium. It was important not to discard these effects because they may have some importance in the detection of delirium. As such it was decided to fit a logistic regression model using Firth's penalized likelihood procedure [50] as coded by Heinze and Schemper [51] in their FL SAS macro. Confidence intervals obtained are then approximate and their coverage probabilities may be larger than usually expected.

## Results

### Description of the study population

Baseline characteristics of the 156 residents are presented in Table 1. The mean age of the participants was 86.3 ± 6.9 (mean ± standard deviation) and the majority were female (73.7%). The mean time since their admission to their LTC facility was 2.6 years (SD = 2.3). The degree of cognitive impairment in this population was important with >95% of residents rated as having severe cognitive decline (*Functional Assessment Staging*) and Alzheimer disease was the most frequent type of dementia. About 41% of the participants had a high level of comorbidity (score ≥ 8 on the *Charlson Comorbidity Index*) and the mean number of prescribed medications was 9.1 (SD = 4.3). More than two third of the residents were showing important deterioration in their functional autonomy (score ≥ 29 on the SMAF).

**Table 1:** Baseline characteristics of residents (N = 156)

**Variables **[Missing values]	**Mean (SD)**	**n (%)**
**Age**	86.3 (6.9)	
65–74		8 (5.1)
75–84		48 (30.8)
≥ 85		100 (64.1)
**Sex**		
Female		115 (73.7)
**Marital status**		
Married-living as married		43 (27.6)
**Level of education **(years) [4]	7.8 (3.5)	
**Ethnicity**		
Caucasian		151 (96.8)
**Days since admission to LTCS**^1 ^[13]	937.3 (838.5)	
**Comorbidity **(CCI) ^2^		
Moderate to severe (≥ 8)		65 (41.7)
**Number of drugs taken**	9.1 (4.3)	
**Types of dementia **[1]		
1. Alzheimer		53 (34.2)
2. Vascular		28 (18.1)
3. Subcortical		7 (4.5)
4. Frontotemporal		0 (0.0)
5. Mixed		22 (14.2)
6. Not specified		45 (29.1)
**Severity of dementia **(FAST) ^3 ^[27]		
Stage 1 to 4		0 (0)
Stage 5 (early dementia)		4 (3.1)
Stage 6 (middle dementia)		93 (72.1)
Stage 7 (late dementia)		32 (24.8)
**Functional autonomy **(SMAF)^4^	34.0 (11.2)	
0–29 (no to mild dependency)		50 (32.0)
29.1–40 (moderate)		51 (32.7)
≥ 41 (severe dependency)		55 (35.3)

1 (LTC) Long-term care facility

2 (CCI) *Charlson Comorbidity Index*

3 (FAST) *Functional Assessment Staging*

4 (SMAF) *Functional Autonomy Measurement System*

Table 2 shows the baseline characteristics of the 40 bedside nurses who took part in the study. All were female with a mean age of 47.3 years (SD = 11.9). The majority of them (56.4%) had a college-degree educational level. The mean number of years of experience in nursing and in geriatrics was 21.2 (SD = 13.3) and 9.9 (SD = 8.3) respectively.

**Table 2:** Baseline characteristics of nurses (N = 40)

**Variables **[Missing values]	**Mean (SD)**	**n (%)**
**Age **[1]	47.3 (11.9)	
≤ 25		3 (7.7)
Between 26–54		21 (53.8)
≥ 55		15 (38.5)
**Gender **[1]		
Female		39 (100.0)
**Level of education **[1]		
College degree		22 (56.4)
Certificate degree (University level)		9 (23.1)
Bachelor's degree		8 (20.5)
**Experience as a nurse **(years) [1]	21.2 (13.3)	
≤ 5		7 (17.9)
6 to 20		12 (30.8)
≥ 21		20 (51.3)
**Experience in geriatrics **(years) [1]	9.9 (8.3)	
≤ 5		16 (41.0)
6 to 20		18 (46.1)
≥ 21		5 (12.8)

### Rates of delirium detection by bedside nurses

In all, 302 paired observations by bedside nurses and RA-1 were made in the 156 residents: 151 at T1 and 151 at T2. Five residents were excluded from the analyses because of missing values. Tables 3 and 4 present the comparison of bedside nurse and RA-1 delirium ratings for each time measurement (T1 and T2).

**Table 3:** Comparison of bedside nurse and researcher (RA-1) delirium ratings at Time 1

	**T1 **(N = 151)	
	
	**RA-1 rating**^1^	
**Nurse rating**	Delirium	No delirium

Delirium	14	3
No delirium	94	40
Sensitivity^2 ^[95% CI] : 13.0% [7.3–20.8]		
Specificity^3 ^[95% CI] : 93.0% [80.9–98.5]		
Positive predictive value^4 ^[95% CI] : 82.4% [56.6–96.2]		
Negative predictive value ^5 ^[95% CI] : 29.9% [22.3–38.4]		

1 Research assistant rating of probable delirium, assessed with the CAM, was used as the reference standard.

2 Indicates how often nurses detected delirium when it was present (true positive rate).

3 Indicates how often nurses rated delirium as absent when it was not present (true-negative rate).

4 Indicates how often delirium was present when delirium was detected by nurses.

5 Indicates how often delirium was absent when delirium was not detected by nurses.

**Table 4:** Comparison of bedside nurse and researcher (RA-1) delirium ratings at Time 2

	**T2 **(N = 151)	
	
	**RA-1 rating**^1^	
**Nurse rating**	Delirium	No delirium

Delirium	20	6
No delirium	87	38
Sensitivity^2 ^[95% CI] : 18.7% [11.8–27.4]		
Specificity^3 ^[95% CI] : 86.4% [72.6–94.8]		
Positive predictive value^4 ^[95% CI] : 76.9% [56.4–91.0]		
Negative predictive value^5 ^[95% CI] : 30.4% [22.5–39.3]		

1 Research assistant rating of probable delirium, assessed with the CAM, was used as the reference standard.

2 Indicates how often nurses detected delirium when it was present (true positive rate).

3 Indicates how often nurses rated delirium as absent when it was not present (true-negative rate).

4 Indicates how often delirium was present when delirium was detected by nurses.

5 Indicates how often delirium was absent when delirium was not detected by nurses.

At T1, the prevalence of delirium was 71.5% (n = 108). Of the 108 residents with delirium, 14 were detected by the bedside nurses, corresponding to a sensitivity of 13%. Alternatively, bedside nurses identified 40 of the 43 cases without delirium, which represents a specificity of 93%. Of the 17 residents rated by the bedside nurses as having delirium, 83% were correctly classified (positive predictive value). However, of the 134 residents evaluated by the bedside nurses as not having delirium, only 29.9% were correctly classified (negative predictive value), suggesting that the absence of a delirium rating by the bedside nurse does not exclude delirium in this specific population.

At T2, RA-1 found evidence of delirium in 107 of the 151 residents (70.9%), thereby indicating practically no variation in the prevalence of delirium between T1 and T2. The comparison of bedside nurse and RA-1 delirium ratings at T2 yielded similar results to those observed at T1. Bedside nurses had difficulty detecting delirium when it was actually present (sensitivity of 18.7%). However, the majority (76.9%) of cases identified by the bedside nurses as having delirium were correctly classified (positive predictive value). Bedside nurses detected most of the cases (86.4%) without delirium but of the 125 cases they identified as not having delirium, only 30.4% of them were correctly classified (negative predictive value).

### Rates of delirium symptoms detection by bedside nurses

Table 5 presents a comparison of the bedside nurse and RA-1 ratings of 6 delirium symptoms (MDS-2 items): easily distracted; periods of altered perception; disorganized speech; periods of restlessness; periods of lethargy; and mental function varies over the course of the day. Sensitivity, specificity and confidence intervals are presented for each symptom. In total, 250 paired observations were made: 125 at T1 and 125 at T2. As previously mentioned, 31 residents were excluded from the analyses because of missing values.

**Table 5:** Validity of Bedside nurse assessments of delirium symptoms compared to RA-1 ratings of delirium symptoms using MDS-2 items^1^

**Symptoms**	**T1 (N = 125)**		**T2 (N = 125)**	
	
	**Sensitivity**^2 ^No. (%) [95% CI]	**Specificity**^3^No. (%) [95% CI]	**Sensitivity **No. (%) [95% CI]	**Specificity **No. (%) [95% CI]
Easily distracted	62/116 (53.4) [44.0–62.8]	6/9 (66.7) [29.9–92.5]	73/117 (62.4) [53.0–71.2]	5/8 (62.5) [24.5–91.5]
Periods of altered perception	9/23 (39.1) [19.7–61.5]	75/102 (73.5) [63.9–81.8]	9/21 (42.9) [21.8–66.0]	78/104 (75.0) [65.6–83.0]
Disorganized speech	50/86 (58.1) [47.0–68.7]	27/39 (69.2) [52.4–83.0]	48/84 (57.1) [45.9–67.9]	28/41 (68.3) [51.9–81.9]
Periods of restlessness	35/108 (32.4) [23.7–42.1]	14/17 (82.3) [56.6–96.2]	29/102 (28.4) [19.9–38.2]	19/23 (82.6) [61.2–95.0]
Periods of lethargy	22/63 (34.9) [23.3–48.0]	47/62 (75.8) [63.3–85.8]	24/57 (42.1) [29.1–55.9]	55/68 (80.9) [69.5–89.4]
Mental function varies over the course of the day	54/118 (45.8) [36.6–55.2]	4/7 (57.1) [18.4–90.1]	56/113 (49.6) [40.0–59.1]	8/12 (66.7) [34.9–90.1]

1 RA-1 ratings of delirium symptoms, assessed with the MDS-2 items, was used as the reference standard.

2 Indicates how often nurses detected delirium symptom when it was present (true positive rate).

3 Indicates how often nurses rated delirium symptom as absent when it was not present (true-negative rate).

At T1, sensitivities ranged from 32.4% to 58.1%, indicating an overall under-recognition of delirium symptoms by bedside nurses compared to the RA-1 ratings for these same symptoms. Nevertheless, 3 symptoms were detected more easily by the bedside nurses: disorganized speech (58.1%), easily distracted (53.4%) and mental function varies during the course of the day (45.8%). The following symptoms were more difficult for the bedside nurses to detect: periods of altered perception (39.1%), periods of lethargy (34.9%) and periods of restlessness (32.4%). Specificities ranged from 57.1% to 82.3%. Periods of restlessness, periods of altered perception and periods of lethargy were the symptoms with the higher specificities (82.3%, 75.8% and 73.5% respectively). These relatively high specificities indicate that these features were rarely identified by nurses when said features were indeed absent. Lower specificities were observed for disorganized speech (69.2%), easily distracted (66.7%) and mental function varies over the course of the day (57.1%) indicating that these features were more often identified as present (compared to the other features) when in reality, they were absent.

At T2, sensitivities and specificities ranged from 28.4% to 62.4% and 62.5% to 82.6% respectively. Despite some differences in the sensitivities and specificities recorded for T2 compared to those of T1, the interpretation of the results remains the same as that described for T1.

### Factors associated with undetected delirium by bedside nurses

Tables 6, 7, and 8 present the results of univariate analyses linking several potential risk factors to the delirium that was undetected by bedside nurses at T1. From among the 18 variables considered, only age was found to be associated with undetected delirium: residents over 85 years old were at greater risk for being undetected (OR: 4.1; 90% CI: [1.5–11.0]).

**Table 6:** Factors associated with undetected delirium (Characteristics of residents).

**Variables**	**T1 **(N = 109)		
	**Delirium detected **[missing values]		**Odds Ratio [90% CI]**

	**No **n = 95 (87.2%)	**Yes **n = 14 (12.8%)	

**Age**			
≥ 85	66 (69.5%)	5 (35.7%)	4.1 [1.5–11.0]^1^
≤ 84	29 (30.5%)	9 (64.3%)	1.0
**Sex**			
Male	22 (23.2%)	6 (42.9%)	2.5 [0.9–6.6]
Female	73 (76.8%)	8 (57.1%)	1.0
**Severity of dementia (HDS)**			
Severe	45 (47.4%)	7 (50.0%)	1.8 [0.6–5.2]
Moderate	32 (33.7%)	2 (14.3%)	4.4 [1.0–19.1]^2^
Mild	18 (19.0%)	5 (35.7%)	1.0
**Behavioural problems (NHBPS)**	[2]		
Yes	44 (47.3%)	8 (57.1%)	0.7 [0.3–1.7]
**Functional autonomy (SMAF)**			
≥ 41	48 (50.5%)	5 (35.7%)	0.6 [0.1–3.6]
29,1–40	30 (31.6%)	8 (57.1%)	0.2 [0.0–1.4]
0–29	17 (17.9%)	1 (7.1%)	1.0
**Number of medications**			
≥ 10	37 (38.9%)	5 (35.7%)	1.7 [0.5–5.2]
6–9	36 (37.9%)	4 (28.6%)	2.0 [0.6–6.7]
≤ 5	22 (23.2%)	5 (37.7%)	1.0
**Pain **(Doloplus – II) Yes(≥ 5)	40 (42.1%)	8 (57.1%)	0.5 [0.2–1.4]
**Depression **(Cornell Scale) Yes (≥ 6)	26 (27.3%)	4 (28.6%)	0.9 [0.3–2.7]
**Sleep problems **Yes (≥ 8)	10 (10.5%)	1 (7.1%)	1.5 [0.3–9.2]
**Visual impairment **Yes	20 (21.1%)	2 (14.3%)	1.6 [0.4–6.0]
**Hearing impairment **Yes	13 (13.7%)	3 (21.4%)	0.6 [0.2–1.9]
**Dehydration **Yes (≤ 500 ml)	33 (34.7%)	6 (42.9%)	0.7 [0.3–1.8]
**Weight loss**	[10]	[2]	
Yes (≥ 3 kg)	16 (18.8%)	3 (25.0%)	1.4 [0.4–4.7]
**Brachial perimeter**		[1]	
**Abnormal **(≤ 21 cm)	15 (15.8%)	2 (15.4%)	1.0 [0.3–4.0]
**Comorbidity **(CCI) Severe (≥ 8)	36 (37.9%)	3 (21.4%)	2.2 [0.7–6.9]
**Geriatric fever**	[7]	[2]	
Yes (T° rectal : ≥ 37,5 ; T° oral : ≥ 37,2)	9 (10.2%)	1 (8.3%)	1.3 [0.2–7.7]
**Oxygen saturation**	[10]	[2]	
Abnormal (<95%)	26 (30.6%)	5 (41.7%)	0.6 [0.2–1.7]

1 Statistically significant p < 0.1

2 The "moderate" category of dementia severity was weakly associated with undetected delirium. However, considering the significance level chosen (0.1) and that the variable "severity of dementia" as a whole was not statistically significant (p = 0.24), it was decided not to consider this association as clinically important.

**Table 7:** Factors associated with undetected delirium (Characteristics of delirium)

	**T1 **(N = 109)		
**Variables**	**Delirium detected **[missing values]		**Odds Ratio [90% CI]**

	**No **n = 95 (87.2%)	**Yes **n = 14 (12.8%)	

**Severity of delirium **(DI)			
Severe	27 (28.4%)	6 (42.9%)	0.6 [0.1–3.7]
Moderate	60 (63.2%)	7 (50.0%)	1.1 [0.2–6.9]
Mild	8 (8.4%)	1 (7.1%)	1.0
**Forms of delirium**	[12]	[2]	
Hypoactive	11 (13.2%)	0 (0.0%)	3.3 [0.5–139.8]^1^
Mixed	28 (33.7%)	6 (50.0%)	0.6 [0.2–1.8]
Without a motor component	6 (7.2%)	1 (8.3%)	0.6 [0.1–4.3]
Hyperactive	38 (45.8%)	5 (41.7%)	1.0

1 In the case of forms of delirium, quasi-complete separation of the data was observed. See statistical analysis details in the method section.

**Table 8:** Factors associated with undetected delirium (Characteristics of nurses)

	**T1 **(N = 109)		
**Variables**	**Delirium detected **[missing values]		**Odds Ratio [90% CI]**

	**No **n = 95 (87.2%)	**Yes **n = 14 (12.8%)	

**Age**	[1]		
≥ 55	30 (31.9%)	6 (42.9%)	0.3 [0.0–2.3]^1^
Between 25–55	57 (60.6%)	8 (57.1%)	0.5 [0.0–3.2]
≤ 25	7 (7.4%)	0 (0.0%)	1.0
**Level of education**	[1]		
Bachelor's degree	19 (20.2%)	1 (7.1%)	3.5 [0.6–20.5]
Certificate degree	20 (21.3%)	3 (21.4%)	1.2 [0.4–3.9]
College degree	55 (58.5%)	10 (71.4%)	1.0
**Experience as a nurse **(years)	[1]		
≥ 21	56 (59.6%)	9 (64.3%)	0.6 [0.1–3.9]
6 to 20	28 (29.8%)	4 (28.6%)	0.7 [0.1–4.9]
≤ 5	10 (10.6%)	1 (7.1%)	1.0
**Experience in geriatrics **(years)	[1]		
≥ 21	17 (18.1%)	6 (42.9%)	0.1 [0.0–0.8]
6 to 20	55 (58.5%)	7 (50.0%)	0.4 [0.1–2.2]
≤ 5	22 (23.4%)	1 (7.1%)	1.0

1 In the case of nurses' age, quasi-complete separation of the data was observed. See statistical analysis details in the method section.

## Discussion

While the detection of delirium by bedside nurses has been studied frequently among hospitalized older patients, this issue has hardly been studied among older residents in LTC facilities. Moreover, as far as the authors are aware, no study to date has addressed the problem of under-recognition of delirium among elderly residents with dementia in LTC facilities.

### Detection of delirium by bedside nurses

The first objective of this study was to determine the rates of delirium detection by bedside nurses. The results have confirmed the importance of the under-recognition of delirium by bedside nurses among this very frail population. Despite the high prevalence of delirium in this cohort (delirium of 71.5% at T1 and 70.9% at T2), nurses were able to identify delirium in only a minority of cases (13% at T1 and 18.7% at T2). However, where nurses did identify delirium, in the majority of cases (93% at T1 and 86% at T2), they were correct. These results underscore how difficult it is for nurses to recognize delirium among this specific population, based solely on their own observations and clinical judgement.

Only one study looking at detection by nurses of delirium among elderly LTC residents was identified in the literature. Culp et al.[19] studied the detection rate of delirium by nurses among LTC residents (n = 37). Contrary to that of the present study, only10% of their cohort was made up of demented residents. Nevertheless, nurses in their study were found to have detected only 26.7% of delirium cases. This relatively higher detection rate compared to that observed in the present study is not surprising since, due to overlapping of some symptoms, delirium superimposed on dementia can make delirium detection more difficult for untrained professionals [5].

Other studies on nurse-detection of delirium among older adults have been carried in hospital settings. Inouye et al. [20] studied nurses' ability to recognize delirium among 797 (>70 years old) hospitalized patients (medical and surgical units). They found an overall sensitivity of 19.3% and specificity of 95.8%. These results are very close to those of the present study. It should be noted that while their study population was not composed exclusively of older patients with dementia, the degree of cognitive impairment in their cohort was substantial (40% of patients having a score of less than 24 on the Mini Mental State Examination) [20]. The reference standard used in Inouye's study was the CAM but the specific algorithm used was not specified (definite vs. probable).

A study by Lemiengre et al. [3] yielded higher sensitivities for detection of delirium by bedside nurses than the ones obtained in the current study. Depending on the method used to measure delirium, different sensitivities were reported: 23.8% for definite delirium and 66.7% for probable delirium. In our study, sensitivities of 13% and 18.7% were obtained with a definition that includes both definite and probable delirium. The higher sensitivities observed in the Lemiengre et al. study compared to those of the present study can probably be explained by differences in the population under study, as well as differences in methodology. For example, contrary to the present study where all participants were demented, only 12% of their study population had a diagnosis of dementia. The greater proportion of dementia in the current study may have complicated the detection of delirium by nurses. As well, the method used by the bedside nurses to measure the presence of delirium differed in the two studies. In the present study, bedside nurses had to respond to only one question seeking to determine if a resident had delirium or not. In Lemiengre's study, bedside nurses had to fill in a form using the CAM algorithm where each delirium feature was illustrated with practical examples. More importantly, the bedside nurses in Lemiengre's study were invited to an hour long information session on delirium and its detection and posters with information about the use of the CAM algorithm were placed in the nursing stations as well. It is quite likely that this intervention had a positive impact on the nurses' ability to detect delirium.

### Detection of the symptoms of delirium by bedside nurses

The second objective of the present study was to identify the delirium symptoms that presented the biggest challenge for nurses to detect. Interestingly, despite an overall under-recognition of individual symptoms of delirium, nurses were better at identifying individual features of delirium than delirium itself, as the higher sensitivities indicate. Disorganized speech (T1:58.1% and T2:57.1%), easily distracted (T1:53.4% and T2:62.4%) and mental function varies over the course of the day (T1:45.8% and T2:49.6%) were more readily recognized by nurses than periods of altered perception (T1:39.1% and T2:42.9%), periods of restlessness (T1:32.4% and T2:28.4%) and periods of lethargy (T1:34.9% T2:42.1%). These results are somewhat similar to those obtained in hospital settings. For example, Morency et al. [21] studied nurses' ability to recognize symptoms of delirium among older patients in acute care settings. They found that perceptual disturbances were detected by bedside nurses in only 41% of the cases, restlessness in 38% and lethargy in 39%. Another study conducted by Lemiengre and colleagues [3] in an acute geriatric ward revealed that it was difficult for nurses to detect fluctuation of symptoms (sensitivity of 45%), inattention (sensitivity of 59%) and disorganized thinking (sensitivity of 61%). Detection rates of delirium symptoms in the Inouye et al. study [20] were relatively lower than either those obtained in the current study or those reported in both Morency's and Lemiengre's studies [3,21]. In fact, they found that nurses were able to recognize inattention in only 15% of the cases and disorganized thinking in only a quarter of the cases. However, the authors did mention that nursing turnover was high in their clinical setting and that the nurses' experience with older patients varied widely. It is likely that these factors explain the sensitivities they observed.

The results of the current study indicate that recognition of delirium and the symptoms of delirium among older LTC residents with dementia is a real challenge for nurses. Interestingly, the rate of detection among this very frail population was generally quite similar to that observed in hospital settings. Three of the key features of delirium, inattention, disorganized thinking and fluctuation of symptoms were more easily detected by nurses. The fact that these results were not reflected by a higher detection rate of delirium itself, suggests that nurses may not have the necessary knowledge about the diagnostic criteria required to define a resident as being delirious. Not only do nurses need to improve their ability to recognize the symptoms of delirium, they also need to develop an awareness of the link between symptoms and the presence of delirium.

### Factors associated with undetected delirium by bedside nurses

The last objective of this study was to identify factors linked to undetected delirium by nurses. From among all the variables considered, only the age of residents was found to be associated; more precisely it was found that delirious residents 85 years of age and over, were 4 times more likely to be undetected than were younger residents.

One possible explanation may be related to the nurses' perception of ageing. In that sense, McCarthy et al. [52] have found that detection of delirium was influenced by the attitude of nurses toward health in ageing. Nurses who regard health in ageing as negative were found to be less likely to detect delirium. However, this assumption still needs to be tested in this specific population.

As far as the present authors are aware, only one study has identified the risk factors for undetected delirium by nurses. Inouye et al. [20] found that an older age (≥ 80 years old) (adjusted odds ratio-OR: 2.8; 95% confidence interval-CI: [1.7–4.7]), the presence of the hypoactive form of delirium (adjusted OR: 7.4; 95% CI: [4.2–12.9]), vision impairment (adjusted OR: 2.2; 95% CI: [1.2–4.0]) and the presence of dementia (adjusted OR: 2.1; 95% CI: [1.2–3.7]) were independently associated with undetected delirium. The current study corroborated the finding that age was an associated factor of undetected delirium.

The strengths of the present study include the quality of the reference standard ratings of delirium: 1) the RA-1 had received intensive training on delirium and its detection by the principal investigator from the study; 2) the presence of delirium was measured using an instrument recognised for its psychometric properties (CAM); 3) ratings of delirium were based on a seven-hour observation period; 4) information was collected in a consistent manner and the involvement of two RAs minimized the possibility of introducing an information bias in the measures of detection of delirium by bedside nurses. As well, 5) it is important to mention that detection rates of delirium were measured twice with a one week time lapse. Conclusions were the same for the two different time measurements. These findings support the internal validity of the study.

This study has several limitations that should be noted. Although the prevalence of delirium in the study population (n = 109) was high, the number of residents with delirium may still have been insufficient to detect a statistically significant association between the variables considered and undetected delirium. Therefore, the lack of significant association in the present study may be an indicator of a lack of power and should not exclude the possibility of these factors playing a role. Another limitation relates to the sampling method utilized. When compared to randomized sampling, convenience sampling, although very practical, may lead to biased results. On the other hand, the design effect was computed and our study found to perform as a simple random designed study. A final limitation concerns the decision to define delirium as meeting the CAM algorithms for definite and probable delirium based on the difficulty of obtaining a valid assessment of the criterion involving the acute onset of symptoms. By so doing, it is possible that some cases of delirium were in fact a type of dementia, e.g. dementia with Lewy bodies is known for its fluctuation feature which can convey a delirium-like picture. Although this information bias may have inflated the prevalence rate of delirium in our study population, it could not have influenced the overall results of this study, especially with regard to nurses' difficulty in recognising the features of delirium.

### Implication for nursing

Given the high prevalence of delirium in LTC settings and the results observed in this study, it is urgent that nurses, who play a key role in the care of these residents, be knowledgeable about delirium. Nursing education, continuing education and new detection tools tailored to demented LTC residents are certainly ways to address the challenge.

Given the aging population, it is critical for newly registered nurses to have both the necessary knowledge and skills to care for elderly adults. However, recent surveys carried out in the U.S and Canada [53,54] have provided evidence of the inadequate gerontological and geriatric content in undergraduate nursing programs for preparing nurses to adequately care for this population. Integrating more geriatric content into the nursing curriculum as well as increasing the number of clinical hours spent on the nursing care of older adults was identified as possible strategies [53,55]. The necessity for nursing schools to commit to the provision of greater gerontology education, as well as the need to increase the number of faculty staff members with expertise in geriatric care, were also identified as key factors in better preparing the next generation of nurses [54].

Continuing education is another important means for improving nurses' knowledge about delirium. Several studies using both formal and informal teaching sessions have yielded encouraging results [56,57]. Interventions exploiting the concept of mentoring have gained increasing importance in assisting nurses to develop best practice care for older patients and this approach has yielded promising results [58]. Based on the findings obtained in the present study, educational programs on delirium should emphasize recognition of those primary features of delirium concerning which nurses showed significant shortcomings (i.e. periods of altered perception, periods of restlessness, and periods of lethargy). In addition, nurse awareness about the link between the presence of symptoms and the diagnosis of delirium also has to be increased.

A further key element in the improvement of delirium detection is the use of screening tools to detect delirium [59]. In addition to being valid and reliable, the screening tool should be easy to administer, impose minimal discomfort in the residents, be integrated into clinical routine and tailored to a demented population. Such instruments now exist for palliative care [60] and the intensive care unit [61]. Future research is needed to develop and validate an instrument with these characteristics among demented residents in LTC settings.

## Conclusion

This study indicated that, among LTC residents with dementia, the detection of delirium and delirium symptoms constitutes a major problem for nurses. Not only do nurses have difficulty recognizing specific symptoms of delirium, they also have difficulty making the necessary connection between the presence of symptoms and delirium itself. These findings suggest that nurses need more education regarding delirium and its detection. This could be achieved by the introduction of educational sessions on delirium along with opportunities for nurses to put into practice their newly acquired knowledge. Implementing mentoring as a professional support strategy for nurses would also be valuable. The importance of the detection of delirium should also be reflected at the organizational level by the adoption of specific procedures and protocols. For example, the fluctuating nature of delirium requires frequent assessments of the resident's cognitive functioning. This is especially important for older individuals with dementia where a change in their cognitive functioning may be more difficult to detect. In this sense, routine clinical assessment is one strategy that could be used to improve detection. Delirium may be the only signal of the onset or exacerbation of a serious physical illness, while failure to diagnose the delirium and adequately treat the underlying disease can result in serious deleterious consequences, even death. Hence, developing ways to increase delirium detection among this very vulnerable population should be a priority.

## Competing interests

The author(s) declare that they have no competing interests.

## Authors' contributions

PV conceived the study and supervised all phases of the study. SR drafted the manuscript and together with PHC, carried out the data analysis. LD was responsible for participant acquisition and data collection. CD managed the data entry and participated in the data collection for the inter-rater tests. All authors read and approved the final manuscript.

## Pre-publication history

The pre-publication history for this paper can be accessed here:


